# Burning mouth syndrome and pelvodynia: A literature review

**DOI:** 10.1097/MD.0000000000032648

**Published:** 2023-01-20

**Authors:** Bérenger Hamon, Marie Orliaguet, Laurent Misery, Sylvie Boisramé

**Affiliations:** a UFR d’Odontologie, University of Western Brittany, Brest, France; b Centre Hospitalier Régional Universitaire de Brest, Brest, France.

**Keywords:** burning mouth syndrome (BMS), chronic pain, pelvodynia, penoscrotodynia, vulvodynia

## Abstract

Burning mouth syndrome (BMS) and pelvodynia are chronic pain still poorly understood and the links between them are all the more so. Health professionals therefore have few resources to understand, diagnose and treat these pains. They may consider and treat these ailments individually, which does not represent optimal care management for the patient and leads to overmedication. This article aims to highlight their contiguity from epidemiological, etiological, diagnostic, and therapeutic perspectives. This study was based on articles which were found using databases such as PubMed and Web of Science. No exclusion criteria were used. Fourteen studies were reviewed. This present work shows that the clinical presentations of these syndromes are similar, as exemplified by their strong association with anxiety and depression. The neurophysiological mechanisms involved in these conditions are similar to those in patients. The diagnosis is essentially based on visual examination and an elimination of all other possible causes. In addition, this work promotes the fact that a common therapy can be implemented when BMS and pelvodynia co-occur. However, the literature on the subject is still very limited. This can be deepened by exploring all the effective treatments in BMS and vulvodynia for penoscrotodynia. Finally, for all these pains, there is a therapeutic order to respect starting with a psychological approach, then topical treatments, systemic therapy and surgical. This therapeutic gradient assists practitioner in their patient’s pain management. This article also allows health care providers to quickly find an effective systemic treatment for a patient with both BMS and pelvodynia.

## 1. Introduction

Burning mouth syndrome (BMS), also referred to as stomatodynia or glossodynia, is defined by the occurrence of chronic and spontaneous pain, which is frequently perceived by patients as an oral burning sensation. Pain is most often localized on the lingual mucosa, but can equally affect other mucosa in the oral cavity.^[1]^

In 2003, Scala et al proposed a distinction between “primary BMS,” which is an essential or idiopathic form of BMS without any somatic cause and “secondary BMS,” which results from a local or systemic pathological condition.^[2]^ Primary BMS is associated with anxiety and depressive disorders in middle-aged women. The prevalence is estimated between 1.5% and 5.1% in the general population.^[3,4]^ Burning sensation can be associated with oral dysesthesia, taste disorders, food hypersensitivity, and xerostomia.^[5,6]^

The International Headache Society categorizes BMS as neuropathic and facial pains in the first edition of the International Classification of Orofacial Pain (2020) and has described it as “An intraoral burning or dysaesthetic sensation, recurring daily for more than 2 hours per day for more than 3 months, without evident causative lesions on clinical examination and investigation.”^[7]^

The pathophysiology of primary BMS is extensively debated but remains poorly understood despite numerous hypotheses attempting to explain its etiopathogenic mechanisms. A recent systematic review demonstrated neuropathic involvement at various levels of neuraxis with the implication of psychological factors, as well as psychiatric disorders, such as anxiety or depression symptoms. Depending on the patient, neuropathic and psychogenic components may exist simultaneously, with a preponderance of one or the other, or may exist individually. These 2 components cannot be dissociated when defining a BMS.^[8]^

BMS is commonly associated with chronic pain outside of the orofacial sphere. Indeed, other “dynias,” which involve similar mechanisms and symptoms, can be found in patients with BMS, especially vulvodynia in women and penoscrotodynia in men as well as pudendal neuralgia, which are grouped together under the term “pelvodynia.” Several authors describe these associations in the literature, but they remain poorly documented.^[5,6,9]^

This study aimed to analyze the literature regarding the association between stomatodynia or BMS and pelvodynia.

## 2. Materials and methods

### 2.1. Research strategy

To inform this literature review, a comprehensive analysis was performed using PubMed and Web of Science databases. The medical subject headings keywords used were stomatodynia and vulvodynia, stomatodynia and penoscrotodynia, burning mouth syndrome and vulvodynia, burning mouth syndrome and penoscrotodynia, glossodynia and vulvodynia, and burning mouth syndrome and pudendal neuralgia.

### 2.2. Selection of articles

Duplicate and irrelevant articles were excluded from analysis. Studies were limited to those adhering to the inclusion criteria for studies suggesting BMS and vulvodynia or penoscrotodynia simultaneously. No exclusion criteria were used. The articles were from French and English languages.

### 2.3. Information extraction

For each included study, the full text was reviewed to extract the epidemiological, etiological, diagnostic, and therapeutic data.

## 3. Results and discussion

### 3.1. Study selection

**Figure 1.**
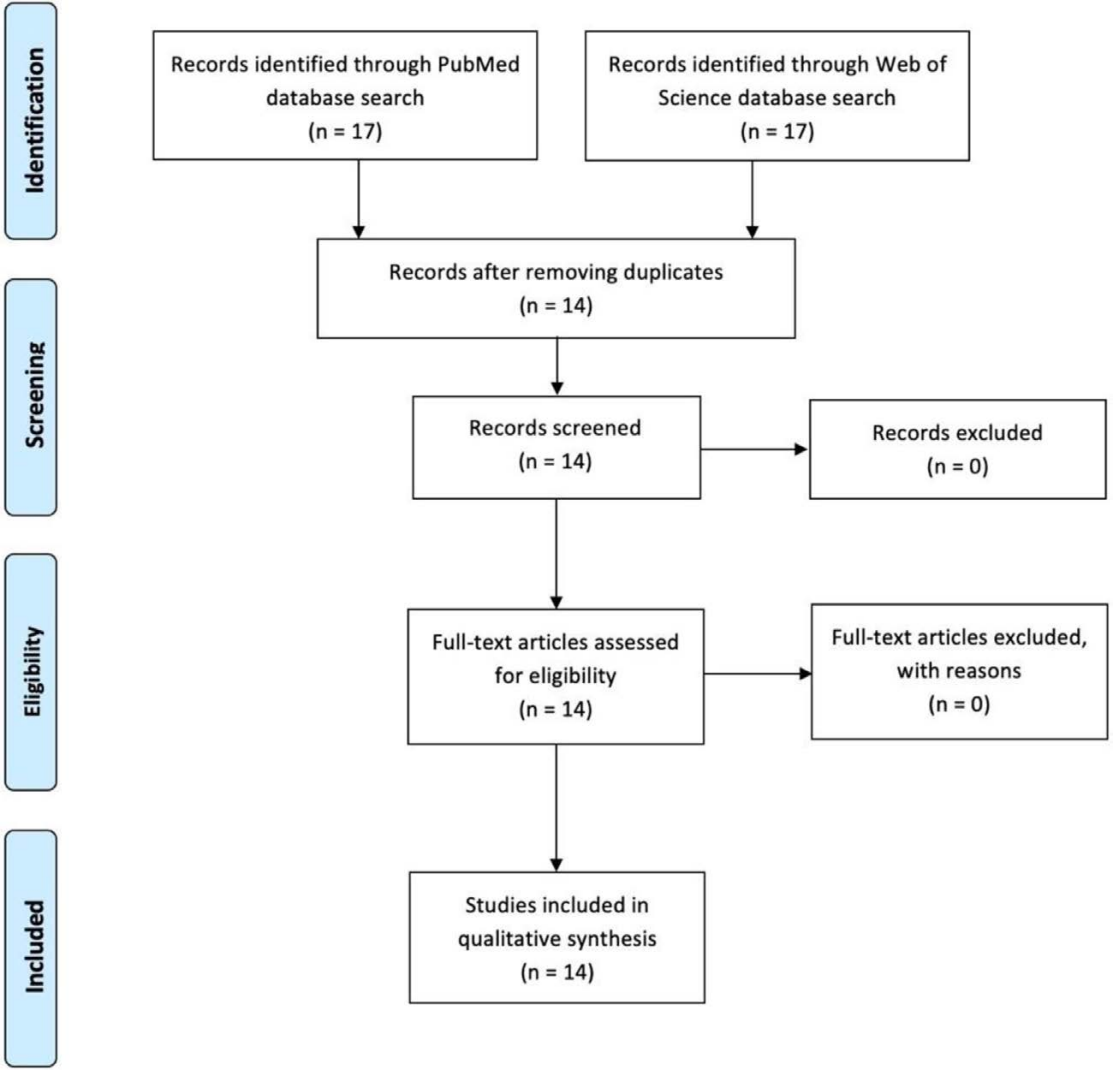
Selection flowchart for articles referring to BMS and pelvodynia. BMS = burning mouth syndrome.

After reading the titles and abstracts, all articles were included. In total, 34 articles were selected: 17 from PubMed and 17 from the Web of Science. After eliminating duplicate or unrelated articles, 14 were included in the data extraction process. The flow diagram in Figure 1 summarizes the study search process. Among the included studies, 9 were review articles,^[1,3,4,10–15]^ 1 was a systematic review^[16]^ and 4 were case reports.^[5,6,9,17]^

### 3.2. Epidemiology

#### 3.2.1. Article origins and patient ethnicities.

All the publications included in this review originated from 2 geographical areas: North America (US, n = 3; Canada, n = 2)^[1,4,10,14,17]^ and Western Europe (France, n = 3; UK, n = 3; Italy, n = 2; Netherlands, n = 1).^[3,5,6,9,11–13,15,16]^

Between 90% and 100% of patients affected by BMS are Caucasian.^[6,14]^ Notably, BMS and pelvodynia have only been studied in developed Western countries and appear to primarily affect Caucasian populations.

#### 3.2.2. Age and chronology of pain.

Patients with BMS were predominantly postmenopausal women in their fifth to seventh decade of life, with an average age of 69 to 73 years. The onset of pain syndrome was more common during the interval of 3 years before and 12 years after menopause.^[3,4,17]^

Patients with vulvodynia are essentially peri-menopausal and postmenopausal. However, some articles reported ages ranging from 20s to the late 60s.^[4,14]^ The co-occurrence of vulvodynia and stomatodynia appears to confirm this information, with a preponderance of patients around the age of 60 years.^[5,6]^

Regarding penoscrotodynia, pain can begin in adolescence as well as in old age, with the majority of patients aged between 35 and 40 years. The co-occurrence of this pain syndrome with BMS was consistent with previous data, with a mean age of 44 years.^[15]^

Globally, the age of onset of BMS and vulvodynia in women is the same. However, penoscrotodynia occurred earlier in men than in those with BMS.

#### 3.2.3. Prevalence.

BMS affects 1.5% to 5.1% of the global population, with a high prevalence in women.^[3,4]^ Eight percent of women were affected by pelvodynia, but only half of them consulted for treatment and only 1.4% had a diagnosis.^[11]^ The lack of data concerning penoscrotodynia in the literature prevents an estimate of its prevalence, but case studies have reported many more cases of penodynia than scrotodynia.^[13]^

Based on 2 cohorts studied, 1.3% to 13.3% of women presented with both BMS and vulvodynia.^[15,16]^

BMS and vulvodynia affected a non-negligible proportion of the population and there was a difference in the number of real chronic pain versus diagnosed chronic pain cases. Data on penoscrotodynia are poorly documented in literature.

#### 3.2.4. Clinical presentation.

The dynias considered in this study often correlated with a history of depression and even more commonly anxiety. While fear of cancer is a redundant trait in chronic pain, patients experiencing chronic pain appear to be in good health.^[4,5,13]^ Only one study described the co-occurrence of genital and oral pain in Parkinson disease.^[17]^

BMS symptoms are burning and/or itching sensations, most often localized on the tongue but capable of extending to the entire oral cavity. They decrease and even disappear with eating and drinking, worsen after swallowing and increase throughout the day. They dramatically impair quality of life and some women describe the symptoms at their peak as worse than those of shingles or childbirth.^[4–6,9,11,14,17]^

The complaints of vulvodynia included pain (70%), burning (64%), tingling (56%), and dyspareunia (58%). The pruritus indicates another disorder.^[4,11]^ These symptoms may be accompanied by an increased urination frequency, vaginal dryness, and dysuria.^[14]^

For penoscrotodynia, a burning sensation and/or pain was described in the penoscrotal region, sometimes with intolerance to the friction of clothing as well as touch, which considerably affects patients’ quality of life.^[9]^ Penoscrotodynia may be associated with red scrotum syndrome, which is a vascular change with hyperemia in the anterior half of the scrotum that may extend to the other half and the base of the penis.^[13]^

Despite different anatomical localizations, patient complaints predominantly share the context of pronounced depression and/or anxiety. This finding supports the hypothesis that BMS, vulvodynia, and penoscrotodynia share common etiological mechanisms, at least in part.

### 3.3. Etiology

#### 3.3.1. Psychogenic factors.

The mechanisms of dissociation and conversion may play a central role in the pathogenesis of cutaneous sensory disorders. Dissociation usually occurs in the context of extreme psychosocial stress and history of severe abuse or neglect early in life. Dissociative patients can experience a sense of detachment from their body and present a state of extreme self-neglect, including the denial of serious skin disorders. Dissociation is characterized by a discontinuity or disturbance in the normal integration of consciousness, identity, memory, bodily representation, perception (involving all senses including sensations originating from the skin), motor control and behavior. Conversion is its somatic component.^[1,10]^

For each of the investigated dynias, the primary patient characteristic was anxiety and depression. Somatization generally refers to bodily symptoms that arise as an expression of an underlying psychological factor or distress. Therefore, somatization is likely to be an important factor in patients with cutaneous sensory disorders and diagnosable psychiatric disorders. For a person who has experienced psychological trauma, this could lead to dissociation (and conversion) mechanisms, turning psychological disorders into physical ones. Literature has reported that the number of traumatic events experienced during a patient’s lifetime is directly related to several idiopathic skin disorders and that the intrusive re-experience of emotional trauma could be experienced as sensory flashbacks to the skin.^[10]^

In the case of vulvodynia, a psychosexual explanation could be that psychological or sexual abnormalities (vestibular mucous hypersensitivity or perineal muscle dysfunction) initially present with sensitization of the central nervous system, leading to allodynia and hyperalgesia. Women with chronic pain are cautious, attentive, pessimistic and vulnerable in their intimate relationships. Therefore, catastrophism in these patients negatively affects their mechanisms of adaptation to pain. Thus, vulvodynia is influenced by both cognitive and affective factors.^[11]^

Notably, many patients with BMS and/or vulvodynia also exhibit psychological fragility. Almost 80% of patients with BMS had alexithymia, which is a personality trait involving dysregulation of negative effect. A history of childhood abuse significantly affected vulvodynia, in that women who were sexually abused in childhood were more likely to develop this condition. Moreover, women with vulvodynia can experience greater pain catastrophizing, namely, a tendency to hold exaggerated negative thoughts and feelings about pain, which can also be described as rumination, helplessness, and magnification.^[8,18,19]^

#### 3.3.2. Neurobiological factors.

The oral cavity, vulvar region and penoscrotal region are characterized by a high density of nerve endings, which makes them ideal sites for the development of cutaneous sensory disorders.^[1,10]^

Regarding the itchy sensation, histamine-specific pruritus transmission neurons have been found in the dorsal horn of the spinal cord, suggesting the existence of a distinct neural pathway from the classic pathways transmitting itchy sensations and which could be involved in itching neuropathic pain.^[10]^

Neurogenic hypotheses include autonomic nervous system disorders, sensory dysfunction associated with sensory fiber neuropathies, disruption of sensory pathways caused by endocrine changes during menopause, disruption of the central nervous system, and modulatory pathways involving the trigeminal nucleus and striatum. Recent advances in this field suggest that BMS is a peripheral sensory small-fiber neuropathy. These findings are based on quantitative sensory testing (QST), which revealed that sensitivity thresholds were disturbed in 76% of patients with BMS.^[3]^ QST is a technique used to evaluate peripheral and central nervous system disorders associated with chronic pain through the administration of calibrated stimuli to measure detection and pain thresholds. Therefore, it can be concluded that the patients presented with somatosensory impairments.

Studies examining BMS using QST have shown that warmth detection and heat pain thresholds can be decreased, suggesting an increase in heat sensitivity in these patients. Conversely, no study has detected a decrease in pain thresholds with the application of cold or mechanical pressure.^[16]^

However, the use of QST in the vulvar region has resulted in decreased pain thresholds for the topical application of acetic acid in women with vulvodynia.^[4]^ Generalized pain sensitivity disorders have also been observed in women with a lowered pressure threshold and pain in the vulva, shoulder, chin and thumbs. These women experience significant increases in the number of pain points, intensity of pain and anxiety induced by pain, which suggests generalized hypersensitivity to pain and pressure.^[11]^ Hyperesthesia in the pudendal dermatome has also been detected in patients with penoscrotodynia, supporting the hypothesis of small-fiber neuropathy.^[13]^

Further research has revealed that the total number of epithelial nerve fibers and innervation density in the fungiform papillae and connective tissue are reduced in patients with stomatodynia associated with axonal degeneration. This finding supports the hypothesis that BMS is a peripheral small-fiber neuropathy.^[3]^

One explanation for vulvodynia is that initial peripheral neuropathy leads to a central sensitization process involving the system, which amplifies pain and causes psychological and sexual problems.

Moreover, in vulvodynia, an increase in interleukin-1β, tumor necrosis factor-α and peripheral nerves has also been observed, which could cause neurogenic regional inflammation. Moreover, patients’ pelvic muscles exhibited increased sensitivity to emotional stimuli. A recent study showed an increase in the number of nociceptive vanilloid receptors in the vestibular region of patients with vulvodynia.^[11]^

Furthermore, an increase in the expression of the vanilloid capscain receptor has been highlighted in the context of BMS, which supports the hypothesis that capscain receptor is involved in the pathophysiology of this dynia.^[20]^

Nevertheless, elements that highlight the neurogenic or psychogenic components should not be opposed. They form 2 complementary explanations: one catalyzes the other in the appearance of symptoms.

### 3.4. Diagnosis

#### 3.4.1. BMS.

Depending on the study, the patients were classified according to the classification of Lamey and Lewis (Table 1), based on the presence or absence of psychiatric disorders, or depending on whether the BMS was primary or secondary.^[3,6,10]^

**Table 1:** Classification of Lamey and Lewis.

Type I	Pain-free waking, with symptoms developing in the late morning and gradually increasing during the day.
Type II	Continuous symptoms throughout the day.
Type III	Intermittent symptoms, with pain-free periods during the day.

Secondary BMS is associated with local or systemic oral causes, such as excessive use of mouthwashes, oral candidiasis, xerostomia, nutritional neuropathy (due to nutritional deficiencies of B vitamins and zinc), dysgueusia, mechanical factors, parafunctional habits, endocrine disorders, psychological disorders, *Helicobacter pylori*, autoimmune disorders, poorly fitting dentures, contact hypersensitivity, and other causes of peripheral neuropathy.^[3]^ Primary BMS was diagnosed after excluding the listed factors. The authors of the examined studies agreed that BMS was primary when no organic cause could explain the pain.^[3,4,6,9,10,17]^

Additional examinations of the visual examination included iron, ferritin, vitamins B1, B6, B12, folate and zinc levels, complete blood count, liver function tests, renal function tests, thyroid function, autoantibody profile (extractable nuclear antigens and antinuclear antibodies), and glycated hemoglobin.

Additional avenues of investigation included candida infection (candida count), hormone imbalance (follicle stimulating hormone, estradiol, thyroid-stimulating hormone, triiodothyronine, and thyroxine) and depending on the clinical history, gastrointestinal disease (antibodies to H. pylori), allergies (serum total IgE patch test for dental materials), and xerostomia (salivary flow rate).^[3]^

#### 3.4.2. Vulvodynia.

Vulvodynia was most often diagnosed by a gynecologist, who ensured that the vulvar area was normal and without any sign of infection or active dermatosis. It is also based on the exclusion of local or systemic factors that might explain pain. These factors were mainly allergens, such as soaps, spermicides, and drugs. The most common sign found on the vulvar mucosa is erythema; however, it is not pathognomonic because it is not present in many asymptomatic women.^[4–6,9–11,14,17]^

A cotton swab or Q-tip test was useful for diagnosis; this test consisted of gently touching all vulvar areas with a cotton swab, starting from the outside and ending with the trigger zones, which are the Bartholin glands.^[11,15]^

Laboratory examinations, such as blood and microbiological samples or biopsies, can be performed to eliminate any systemic or local cause.^[4,9]^

#### 3.4.3. Penoscrotodynia.

The authors classified penoscrotodynia according to either the intensity of pain (severe, moderate, or mild) or mode of onset (provoked, unprovoked, or mixed). The diagnosis of penoscrotodynia was also based on burning sensations and/or pain in the penoscrotal region without an apparent cause detected by visual examination. Erythema was frequently observed in the patient. Therefore, it is necessary to eliminate all secondary causes, such as infection, tumor, testicular torsion, variocele, hydrocele, spermatocele, or a history of surgery or allergens such as soaps.

Performing penoscopy or biopsy is not reasonable if the skin looks healthy.^[4,13]^

It is important to emphasize that primary BMS, vulvodynia and penoscrotodynia are diagnosed based on exclusion. A diagnosis of chronic pain can only be made after eliminating all possible local and systemic causes that could lead to a differential diagnosis. The first step consisted of visual examination of erythema. This initial examination can be deepened by laboratory examinations or even biopsies, if this option remains reasonable.

### 3.5. Treatments

#### 3.5.1. Psychological approach.

All authors agreed that it was necessary to recognize and name these pain syndromes in order to reassure patients. It is important for patients to understand that their symptoms are not indicative of a serious underlying disease. It would then be relevant to invite the patient to consult a psychologist or psychiatrist, preferably trained in pain management.^[4]^ Several authors agree on the need to resort to cognitive behavioral therapy in the context of BMS or pelvodynia because of the significant psychogenic component of these pains.^[3,4]^ In addition, patients could also consult a sex therapist.^[4,11]^

In many instances, there is an underlying condition of anxiety or depression that must be treated before treating the symptoms to obtain lasting results. A Swedish study noted that this psychological approach would improve quality of life in 79% of patients.^[21]^

#### 3.5.2. Local treatments.

Several local treatments have been used in patients with BMS or pelvodynia with varying degrees of success. In some cases, the application of topical anesthetics, such as lidocaine ointments, to the painful sites was slightly relieved but did not eliminate the symptoms and helped improve the patient’s sexual life. Ointments based on corticosteroids such as beclometasone dipropionate are useless or even dangerous in the absence of a diagnosis of progressive inflammatory dermatosis. In addition, local antibacterial, antifungal, and antiviral agents seemed to be ineffective.^[9,11,13,14]^

In BMS, mouthwashes with clonazepam are effective.^[6]^ Some authors have recommended desensitization to capsaicin using mouthwashes containing hot peppers diluted in water. Potential recommendations for patients with pelvodynia include wearing loose clothing, washing with mild soaps, or eliminating detergents by rinsing clothes twice. Transcutaneous electrical nerve stimulation (TENS) might relieve pain in patients with penoscrotodynia. For vulvodynia, some authors have recommended local estrogen therapy, whereas others have suggested vaginal physiotherapy with biofeedback. The results of the latter ranged from 71.4% to 85.7% of patients, indicating an improvement in symptoms. A final option of injecting botulinum toxin into the bulbospongiosus muscles demonstrated significant results at 3 and 6 months.^[4,11,14]^

The results of all aforementioned treatments correspond to the latest scientific data on local treatments. However, they can be supplemented with capsaicin desensitization and low-level laser therapy, which involves applying a low-power laser to areas of vulvodynia pain. These latest therapies have shown encouraging results and can be explored in the context of penoscrotodynia. Furthermore, TENS can be used to treat vulvodynia. Finally, some authors have proposed alternative treatments, such as acupuncture or hypnosis, but more studies are needed to prove their effectiveness.^[13,18,22,23]^

#### 3.5.3. Systemic treatments.

**Figure 2.**
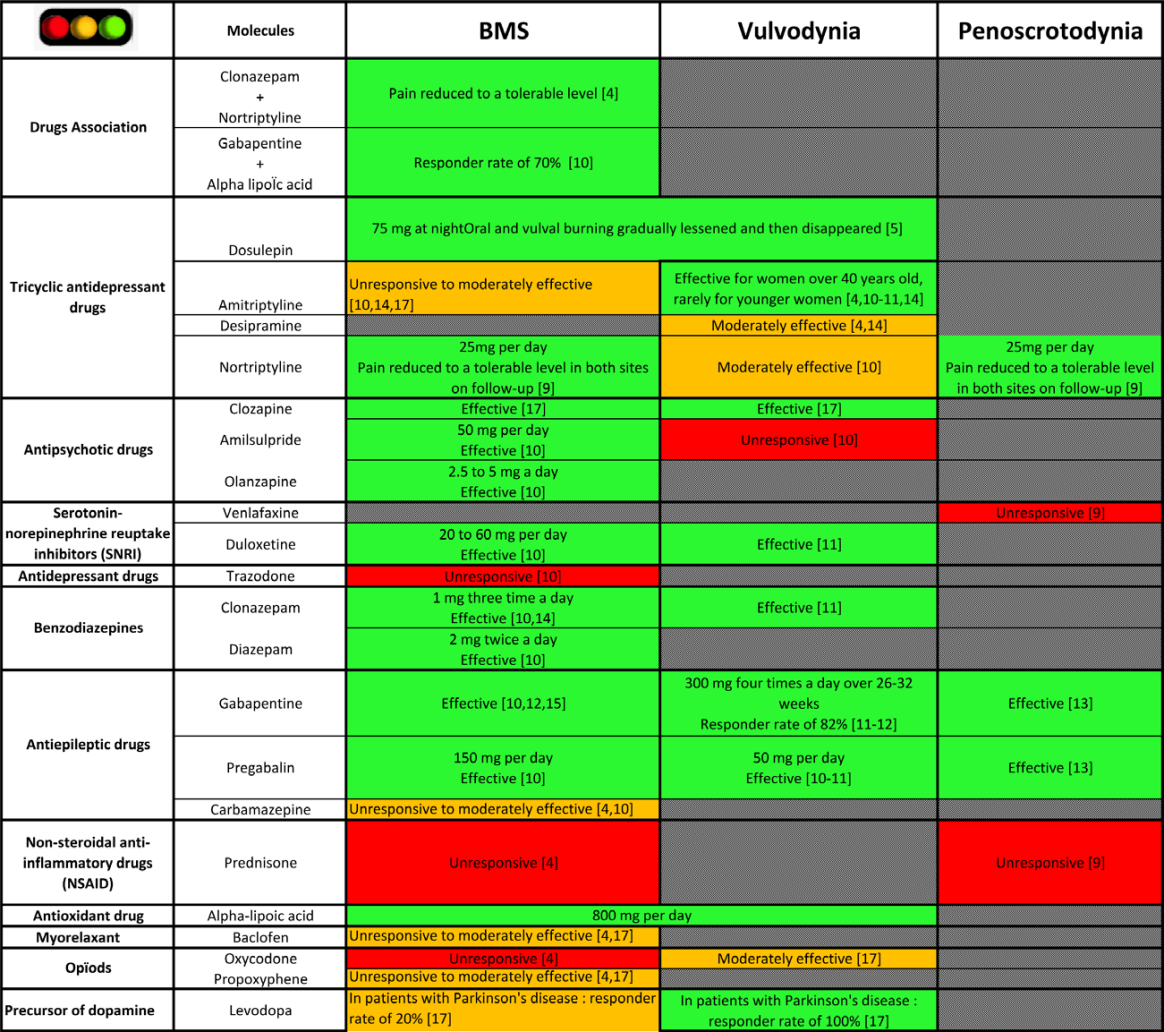
Effectiveness of systemic treatments on BMS, vulvodynia and penoscrotodynia. Green boxes indicate an effective treatment, orange boxes indicate a moderately effective treatment and red boxes indicate an ineffective treatment. BMS = burning mouth syndrome.

Figure 2 depicts the efficacy of systemic treatment for BMS, vulvodynia and penoscrotodynia. Systemic treatments that have shown efficacy in patients with these conditions are gabapentin (1200 mg/day for over 26–32 weeks) and pregabalin (50–150 mg/day).^[10–14]^ Therefore, these are the therapies of choice for patients with BMS and pelvodynia. However, some treatments that succeed in BMS and vulvodynia, such as dosulepin (75 mg/day), α-lipoic acid (800 mg/day), clozapine, duloxetine (20–60 mg/day), and clonazepam (3 mg/day), could be used for patients who have a co-occurrence of these 2 pain syndromes.^[5,6,10,11,14,17]^ These treatments have yet to be studied in patients with penoscrotodynia. None of the selected studies explored whether drugs effective for BMS, such as the combination of gabapentin and nortryptiline or gabapentin and α-lipoic acid, diazepam and olanzapine, were also effective for pelvodynia.^[4,10]^ The remaining treatments covered in the studies (i.e., amitriptyline, desipramine, carbamazepine, levodopa, prednisone, baclofen, oxycodone, and propoxyphene) did not appear to effectively improve symptoms.^[4,10,11,14,17]^

An association between α-lipoic acid and gabapentin or pregabalin is a concept that clinical studies have proven relevant.

#### 3.5.4. Surgical treatments.

Surgical treatment can be envisaged only in the context of pelvodynia. The response rate for vestibulectomy is 61% to 94%. However, there is no evidence that orchiectomy or epididymectomy improves the pain. In contrast, testicular denervation presents encouraging results.^[4,11,13]^

### 3.6. Synthesis

Based on these results, a therapeutic gradient can be established for treating patients with BMS and pelvodynia. First, it is essential to prescribe CBT to patients cognitive behavioral therapy. The next step is to administer local treatments such as lidocaine-based ointments, painful sites, TENS sessions and/or low level laser therapy sessions. If these measures are insufficient and the pain still affects the patient’s quality of life, it is possible to prescribe systemic treatment, beginning with gabapentin or pregabalin. If a patient remains unsatisfactory, the benefit of surgery must be assessed, primarily in the case of vulvodynia.

## 4. Conclusion

Although BMS, vulvodynia and penoscrotodynia (pelvodynia) affect different organs, they share some similarities. First, from an epidemiological perspective, BMS and vulvodynia occur at similar ages and have similar clinical presentations. Similar neurobiological and psychogenic mechanisms have been implicated in this etiology. Neurobiological and psychogenic factors complement the pathophysiology of pelvodynia. Diagnostically, all of these conditions were based on exclusion criteria.

This article highlights common treatments that are valuable in the case of co-occurrence of BMS and pelvodynia, which combine cognitive behavioral therapy with local and systemic treatments, and pregabalin and gabapentin, which have proven effective against all types of pain discussed. However, the effectiveness of drugs such as dosulepin, α-lipoic acid, duloxetine, and clonazepam remains unclear in the context of penoscrotodynia because of the lack of documentation of this chronic pain and merits clinical trials to complete the therapeutic arsenal provided in this article.

## Author contributions

**Analysis of papers:** B. Hamon.

**Bibliographical research:** B. Hamon.

**Conceptualization:** S. Boisrame.

**Methodology:** S. Boisrame.

**Project administration:** S. Boisrame.

**Selection of papers:** S. Boisrame, B. Hamon.

**Supervision:** S. Boisrame.

**Validation:** S. Boisrame.

**Writing – original draft:** B. Hamon, S. Boisrame.

**Writing – review & editing:** B. Hamon, M. Orliaguet, L. Misery, S. Boisrame.
